# Structural Characterization of Neutral and Acidic Glycolipids from *Thermus thermophilus* HB8

**DOI:** 10.1371/journal.pone.0035067

**Published:** 2012-07-16

**Authors:** Yasuo Suda, Fumiaki Okazaki, Yushi Hasegawa, Seiji Adachi, Koichi Fukase, Susumu Kokubo, Seiki Kuramitsu, Shoichi Kusumoto

**Affiliations:** 1 Department of Chemistry, Biotechnology and Chemical Engineering, Graduate School of Science and Engineering, Kagoshima University, Kagoshima, Japan; 2 RIKEN Harima Institute at SPring-8, Sayo-gun, Hyogo, Japan; 3 Department of Chemistry, Graduate School of Science, Osaka University, Toyonaka, Osaka, Japan; 4 Department of Biology, Graduate School of Science, Osaka University, Toyonaka, Osaka, Japan; Imperial College London, United Kingdom

## Abstract

The structural characterization of glycolipids from *Thermus thermophilus HB8* was performed in this study. Two neutral and one acidic glycolipids were extracted and purified by the modified TLC-blotting method, after which their chemical structures were determined by chemical composition analysis, mass spectrometry (MS) and nuclear magnetic resonance (NMR) spectroscopy. The structure of one of the neutral glycolipids, NGL-A, was Gal*p*(α1-6)Glc*pN*acyl(β1-2)Glc*p*(α1-)acyl_2_Gro, and the other, NGL-C, was Gal*f*(β1-2)Gal*p*(α1-6)Glc*pN*acyl(β1-2)Glc*p*(α1-)acyl_2_Gro. The structure of NGL-C was identical to that reported previously [Oshima, M. and Ariga, T. (1976) *FEBS Lett*. **64**, 440]. Both neutral glycolipids shared a common structural unit found in the *Thermus* species. The acyl groups found in NGL-A and NGL-C, iso-type pentadecanoxy and heptadecanoxy fatty acid, were also the same as those found in this species. In contrast, the acidic glycolipid, AGL-B, possessed the structure of *N*-(((Glc*pN*Ac(α1-)acyl_2_Gro)P-2)GroA)alkylamine. The alkyl group in AGL-B was an iso-type heptadecanyl, suggesting that the iso-type structure of the long alkyl chain is responsible for the thermal stability of the bacteria.

## Introduction

*Thermus thermophilus* HB8 is an aerobic, rod-shaped, non-sporulating, Gram-negative eubacterium, which can grow at temperatures over 75°C [1]. *T. thermophilus* is the most thermophilic bacterium whose gene manipulating system has been established among thermophilic bacteria. The number of genes (ORF) is about 1/15 of humans, and the produced proteins are heat-stable and easily crystallized, so are suitable for detailed physicochemical analyses [2]–[4]. Therefore, this bacterium is being used for the “Structural-Biological Whole Cell Project”, which aims to understand all biological phenomena necessary for cell life based on the structural information of biomolecules [5], [6].

From another point of view, since this bacterium can grow under severe circumstances, it may contain a unique chemical structure on its cell surface. In addition, since *T. thermophilus* has a very thin cell wall structure compared to regular bacteria, assembly of cell surface components, including glycolipids, may work as an important structural unit in *T. thermophilus*. In this paper, we focused on cell surface glycolipids, which were extracted with chloroform/methanol at room temperature, and analyzed their structural characteristics by MS and NMR. The structures elucidated were compared to glycolipids from *Deinococcus radiodurans* [7]–[9], which is a Gram-negative and radiation-resistant bacterium possessing genomes similar to those of *T. thermophilus* [10].

## Methods

### Bacterium and Extraction of Glycolipids

*Thermus thermophilus* HB8 cells were grown at 75°C under moderate aeration in a medium containing 0.5% yeast extract, 1% polypeptone, and 1% NaCl at pH 7, and frozen at –80°C until use. The frozen cells (50 g) were thawed and suspended in 100 ml of distilled water. To the suspension 200 ml of methanol and 110 ml of chloroform were added, and stirred for 90 min at room temperature. After centrifugation (5150 g, for 30 min), the supernatant was collected and concentrated with a rotary evaporator, and lyophilized to obtain crude extract (1.2 g).

### Instruments

Gas chromatography (GC) was performed with GC-14BPF 100 V (Shimadzu, Kyoto, Japan) with FID as a detector. GC/MS analysis was done using a GC-17A/QP-5000 (Shimadzu). MALDI-TOF/MS was performed with a Voyager RP-DE (PerSeptive Biosystems, Framingham, MA, USA) using a 2, 5-dihydroxybenzoic acid (DHBA) as a matrix. ESI-TOF/MS was done using a Mariner™ (PerSeptive Biosystems). NMR analysis was performed with a JNM-LA 500 spectrometer (JEOL, Tokyo, Japan), an Excaliber-400 spectrometer (JEOL) or a UNITY 600 plus spectrometer (Varian, Palo Alto, CA, USA) at 303 K. Trimethylsilane (TMS) or methanol was used for the internal standard in the ^1^H and ^13^C spectra.

**Table 1:** MS analyses of neutral and acidic glycolipids from *T. thermophilus* HB8.

	NGL-A	NGL-C	AGL-B
Mode	Positive	Positive	Negative
Detected Ion	[M+Na]^+^	[M+Na]^+^	[M-H]^-^
Intact	1356.8	1519.0	1204.0
m/z	1328.7	1491.1	1176.0
	1300.7	1463.0	
	1270.7	1434.1	
Per-acetylated	1734.7	2023.7	1329.9
Compound	1706.6	1995.7	1302.0
m/z	1678.6	1965.7	
	1648.7	1937.6	
No. of acetyl groups incorporated by per-acetylation	9	12	3

### Analytical Procedures

Phosphorous contents were determined according to the method of Bartlett [11]. Hexose content was measured by the anthrone-sulfuric acid method [12]. Analysis of the sugar constituents of the sample was performed by the alditol acetate method [13]. Glycerol and hexosamine were analyzed as described previously [14]. Fatty acids were analyzed according to the method of Ikemoto *et al*. [15].

NMR spectra of the glycolipids were obtained at 303 K at a concentration of 0.6 mg/ml in CDCl_3_-CD_3_OD (2/1 or 1/1, v/v) or in CDCl_3_. The chemical shifts are expressed in δ-values using trimethylsilane (δ 0) for the ^1^H spectra and methanol (δ 48.90) for the ^13^C spectra. One-D DANTE, DQF-COSY, TOCSY, ROESY, HMQC, and HMBC spectra were performed according to the method described [16].

In MALDI-TOF/MS measurements, samples were dissolved in chloroform-methanol (1/1, v/v), mixed with saturated 2,5-dihydroxybenzoic acid (DHBA) dissolved in the same solvent mixture as a matrix, and placed on a sample plate. Spectra were obtained using the RDE1000 method. In the ESI-TOF/MS, the sample was dissolved in dichloromethane/methanol (1/1, v/v) at a concentration of 1 µg/ml and introduced at a flow rate of 1 µl/min.

**Table 2:** Chemical Composition of neutral and acidic glycolipids extracted from *T. thermophilus* HB8.

	NGL-A		NGL-C		AGL-B	
	µmol/mg	(molar ratio)	µmol/mg	(molar ratio)	µmol/mg	(molar ratio)
Phosphate	−		−		0.77	(1.0)
Fatty acid		(2.5)		(3.1)		(2.0)
C15∶0(13-Me)	0.24	(0.5)	0.13	(0.4)	0.29	(0.4)
C15∶0(12-Me)	0.07	(0.1)	0.09	(0.3)	0.05	(0.1)
C16∶0	0.11	(0.2)	−	−	0.09	(0.1)
C17∶0(15-Me)	0.70	(1.3)	0.48	(1.4)	0.91	(1.1)
C17∶0(14-Me)	0.17	(0.3)	0.33	(1.0)	0.22	(0.3)
C18∶0	0.04	(0.1)	0.01	(0.0)	−	−
Sugar						
Glycerol	0.46	(0.9)	0.39	(1.1)	0.51	(0.7)
Galactose	0.65	(1.2)	0.91	(2.7)	−	−
Glucose	0.65	(1.2)	0.45	(1.3)	−	−
Glucosamine	0.53	(1.0)	0.34	(1.0)	0.80	(1.0)

Molar ratio was calculated based on glucosamine.

### Thin Layer Chromatography (TLC) and TLC-blotting

Thin layer chromatography was performed using a silicagel plate (Merck Silicagel 60F254 No. 5715). The crude extract was dissolved in chloroform/methanol (1/1, v/v) and spotted on the plate, then developed with chloroform/methanol/water/triethylamine (30/8/0.7/0.01, v/v), or chloroform/methanol/water/acetic acid (13/1/1/5, v/v). Separated compounds were visualized with anisaldehyde-sulfuric acid or water.

Isolation of separated glycolipids was performed by the TLC blotting method [17] with an effective modification. In brief, crude extract was first separated by TLC as above and marked with a pencil. The TLC plate was soaked in blotting buffer (chloroform/methanol/1.5 M ammonium bicarbonate, 40/30/7, v/v) for 10 sec. Two PTFE (polytetrafluoroethylene) membranes presoaked in the above blotting buffer were placed on the TLC plate. The plate and membranes were pressed with glass and subjected to preheated thermal blotter (ATTO, Tokyo, Japan). The press (6.4 kg/plate) with the blotter continued for less than 30 sec at 180°C. By repeating the treatment (regularly 3 to 4 times) and changing the PTFE membrane, all of the separated glycolipids were transferred to the membrane from the TLC plate. The membranes were then soaked in chloroform/methanol (1/1, v/v) to release the separated glycolipids. The solution was washed with water to remove the acetic acid, concentrated and lyophilized with water to obtain the purified glycolipids. From 180 mg of crude glycolipid, three kinds of glycolipids (named NGL-A, NGL-B, and AGL-B) were isolated in yields of 23 mg, 18 mg and 65 mg, respectively.

**Table 3:** NMR data of per-acetylated neutral glycolipids extracted from *T. thermophilus* HB8.

	Per-acetylated NGL-A		Per-acetylated NGL-C	
	^1^H	^13^C	^1^H	^13^C
	Chemical shift δ (^3^ *J*_H,H_, Hz)	chemical shift δ	Chemical shift δ (^3^ *J*_H,H_, Hz)	chemical shift δ
Glycerol				
Gro-1	4.41, dd(3.1, 12.1); 4.35 dd(7.4, 12.2)	63.6	4.40, dd (3.1, 8.0); 4.33 dd(7.4, 12.4)	63.3
Gro-2	5.30-5.29, m	69.8	5.28-5.24, m	69.7
Gro-3	3.90, dd(5.0, 11.1); 3.78, dd(4.1, 11.2)	67.9	3.90, dd(5.5, 11.2); 3.78, dd(4.2, 11.3)	67.6
Glucose				
Glc-1	4.88, d(3.7)	99.3	4.86, d(3.6)	99.1
Glc-2	3.94, dd(3.7, 10.0)	73.9	3.93, dd(3.6, 11.2)	74.3
Glc-3	5.39, dd(9.7, 9.7)	72.1	5.39, dd(9.7, 9.7)	71.7
Glc-4	4.99, dd(9.8, 9.8)	69	5.03, dd(7.8, 8.5)	69.3
Glc-5	4.03-3.99, m	67.8	4.02-3.99, m	68
Glc-6	4.25, dd(5.0, 12.3); 4.05, dd(2.3, 12.3)	62.3	4.19, m; 4.05-4.03, m	62.7
Glucosamine				
GlcN-1	5.32, d(8.2)	98.6	5.33, d(8.1)	98.9
GlcN-2	2.96,m	57.5	3.08, m	57.2
GlcN-3	5.82, dd(9.0, 10.7)	70.8	5.82, dd(9.0, 10.5)	70.6
GlcN-4	4.98, dd(9.2, 9.2)	69.7	4.83, dd(9.4, 12.3)	70.7
GlcN-5	3.70, m	65.2	3.78-3.75, m	72.7
GlcN-6	3.68-3.63, m	73.2	3.76, d(5.3); 3.61, d(7.4)	67.4
GlcN-NH	6.27, d(7.2)	-	6.24, d(7.2)	-
Galactose				
Gal*p*-1	5.25, d(3.6)	96.3	4.97, d(3.4)	98.3
Gal*p*-2	5.03,dd(3.6, 10.9)	68.4	3.96, dd(3.4, 11.7)	73.2
Gal*p*-3	5.34, dd(3.3, 10.9)	67.4	5.32, dd(3.4, 10.7)	68.4
Gal*p*-4	5.44, dd(1.0, 3.3)	68.1	5.44, dd(3.4, 9.4)	68.3
Gal*p*-5	4.21-4.18, m	66.6	4.21, m	66.8
Gal*p*-6	4.16-4.06, m	61.7	4.20-4.06, m	61.4
Galactose				
Gal*f*-1	−	−	5.14, s	98.3
Gal*f*-2	−	−	5.08, dd(0.8, 2.4)	73.2
Gal*f*-3	−	−	5.04, dd(5.7, 10.2)	68.4
Gal*f*-4	−	−	4.28, dd(1.7, 6.1)	68.3
Gal*f*-5	−	−	5.38-5.36, m	66.8
Gal*f*-6	−	−	4.32, dd(4.9, 13.3)	61.4
			4.24, dd(7.1, 12.0)	
Acyl				
1	−	174.3; 173.9; 173.3	−	174.0; 173.7; 173.1
2	2.30, t	36.5; 34.5; 34.3	2.35-2.31, m	34.4; 34.4; 34.2
3	1,55, m	25.4	1.55, m	25
4	1.35-1.25, m	28.1	1.35-1.25, m	28
5∼12(10)	1.35-1.25, m	30.1-29.2		30.1-29.1
13(11)	1.35-1.25, m	27.5		27.5
14(12)	1.14, m	39.2	1.15, m	39.1
15(13)	1.62, m	25.1; 25.0	1.62, m	25.1, 25.0
16, 17(14, 15)	0.86, d(6.6)	22.7	0.86, d(6.6)	22.7

The spectra were measured in CD_3_OD at 303 K. The assignments were established by ^1^H and ^13^C one-dimensional spectroscopy and two-dimensional methods (COSY, TOCSY, HMQC and HMBC).

### Chemical Modification

#### Peracetylation

Peracetylation was performed by the pyridine/acetic anhydride method. In brief, a glycolipid, NGL-A (1.12 mg), was dissolved in pyridine (1.0 ml)/acetic anhydride (0.5 ml), and stirred overnight at room temperature. After removing the solvent, chloroform (1.0 ml) and distilled water (1.0 ml) were added to the residue. After the extraction, the organic phase was removed and evaporated. The residue was applied to silicagel column chromatography (Silicagel Merck No. 9385, 1.0 g, solvent: chloroform/acetone 1/1→10/1, v/v) to obtain 1.13 mg of peracetylated NGL-A. Yield 78.5%.

**Figure 1:**
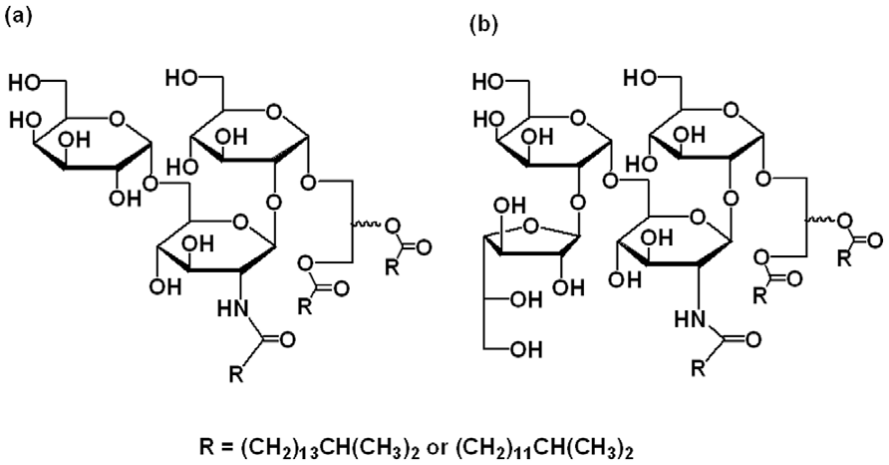
Proposed structures of neutral glycolipids from *T. thermophilus* HB8. (a) NGL-A; (b) NGL-C.

**Figure 2:**
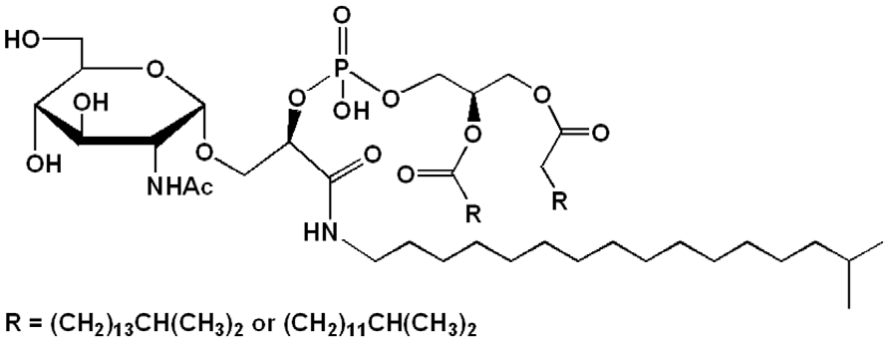
Proposed structure of the acidic glycolipid (AGL-B) from *T. thermophilus* HB8.

#### Alkali-hydrolysis (methanolysis) and subsequent acetylation

A purified glycolipid (AGL-B, 4.7 mg) was dissolved in 1 ml of methanol. To the solution, 0.1 ml of 1 M methanol solution of sodium methoxide was added dropwise at 0°C. After stirring for 30 min at 0°C, the pH of the solution was neutralized by the addition of Dowex 50-X8 (H). After the removal of the resin by filtration, water was added to the filtrate. Extraction with chloroform was done 3 times, after which the chloroform was evaporated and water was added to the residue. After lyophilization of the resulting suspension, the obtained white powder was dissolved in methanol. To remove fatty acids, extraction was done with hexane. The residual methanol was evaporated to obtain a white powder, which was then dissolved in pyridine (1 ml)/acetic anhydride (0.5 ml). The reaction solution was stirred for 1 h at room temperature, and concentrated to dryness. Water was added to the residue, and the resultant suspension was lyophilized to obtain a white powder (4.3 mg).

#### Modified Mosher method

Similar alkali hydrolysis of the purified glycolipid (AGL-B, 2.5 mg) was performed using sodium methoxide in methanol. The hydrolyzed product, glyceramide derivative, was purified by silicagel chromatography and confirmed by ESI-TOF/MS (m/z 569.41 [M+Na]^+^) and NMR. Then, the modified Mosher method was applied to the glyceramide derivative according to the literature [18]. In brief, the purified glyceramide (0.51 mg) was dissolved in 0.1 ml of pyridine, and (+)-MTPA (α-methoxy-α-(trifluoromethyl)phenylacetic acid) chloride (6.8 mg, 27 µmol) was added at 0°C. After stirring for 22 h at room temperature, the reaction mixture was concentrated. Then, the obtained residue was applied to silica gel column chromatography (elution: hexane/ethyl acetate  = 1/1) to isolate the tri-(R)-MTPA ester derivative (0.83 mg, confirmed with the positive mode of ESI-TOF/MS: m/z 1195.52 [M+H]^+^) as a colorless solid. Using (–)-MTPA chloride, the tri-(S)-MTPA ester derivative was obtained similarly.

**Table 4:** NMR data of alkali-hydrolyzed and per-acetylated products of the acidic glycolipid (AGL-B) from *T. thermophilus* HB8.

	^1^H	^13^C
	Chemical shift δ (^3^ *J*_H,H_, Hz)	chemical shift δ
Glucosamine		
GlcN-1	4.87, d(3.4)	98.0
GlcN-2	4.28, dd(3.3, 10.5)	51.8
GlcN-3	5.14, m	71.3
GlcN-4	5.11, m	68.1
GlcN-5	3.92, m	68.4
GlcN-6	4.23, dd(4.4, 12.4); 4.10, dd(1.3, 12.4)	61.9
GlcN-NH	6.03, d(9.4)	−
GlcN-3COCH_3_	−	171.4
GlcN-3COCH_3_	2.01, s	20.8
GlcN-4COCH_3_	−	169.3
GlcN-4COCH_3_	2.01, s	20.8
GlcN-6COCH_3_	−	170.8
GlcN-6COCH_3_	2.10, s	20.7
GlcN-NCOCH_3_	−	170.2
GlcN-NCOCH_3_	1.97, s	23.2
Glyceroyl		
GloA-1	−	166.8
GloA-2	5.39, dd(3.3, 5.6)	72.8
GloA-3	4.03, dd(5.7, 11.5); 3.85, dd(3.4, 11.8)	67.8
GloA-2COCH_3_	−	169.6
GloA-2COCH_3_	2.22, s	21.1
Alkylamide		
RN-NH	6.16, t(5.7)	−
RN-1	3.31-3.27, m	39.7
RN-2	1.54, m	29.8
RN-3	1.31, m	27.0
RN-4∼12(10)	1.30-1.26, m	30.1-29.6
RN-13(11)	1.31, m	27.0
RN-14(12)	1.15, m	39.2
RN-15(13)	1.54, m	29.6
RN-16,17(14,15)	0.86, d(6.6)	22.8

The spectra were measured in CDCl_3_ at 303 K. The assignments were established by ^1^H and ^13^C one-dimensional spectroscopy and two-dimensional methods (COSY, TOCSY, HMQC and HMBC).

#### Phospholipase A_2_ digestion

According to the literature [19], the purified glycolipid (AGL-B, 1.1 mg, 0.93 µmol) was dissolved in 0.1 ml of diethylether, and 0.1 ml of 0.05 M Tris-HCl buffer (pH 8.9) containing 5 mM CaCl_2_, 0.15 M NaCl and phospholipase A_2_, type I (Sigma Chem. Co., St. Louis, MO) was added. After stirring for 3 h at room temperature, the reaction solution was concentrated. Then, the obtained residue was applied to silica gel column chromatography (elute with ethyl acetate/methanol  = 2/1, v/v) to separate the deacylated compound as a colorless solid. Negative mode ESI-TOF/MS: m/z 951.61 [M-H]^−^.

## Results

### TLC and TLC-blotting

Three kinds of glycolipids existed in the crude extract from *T. thermophilus*. In the TLC analysis, when the solvent was changed to chloroform/methanol/water/acetic acid from chloroform/methanol/water/triethylamine, one component (component B) moved rapidly, although the other two components (A and C) moved similarly. From this fact, it was suggested that the component B was an acidic glycolipid (abbreviated as AGL-B), and the other two were neutral ones (abbreviated as NGL-A and NGL-C).

**Table 5:** NMR data of per-acetylated acidic glycolipid (AGL-B) from *T. thermophilus* HB8.

	^1^H	^13^C	^31^P
	Chemical shift δ (^3^ *J*H,H, Hz)	Chemical shift δ	Chemical shift δ
Glucosamine			
GlcN-1	4.85, d(3.2)	98.9	
GlcN-2	4.28, dd(3.3, 10.5)	53.4	
GlcN-3	5.18, dd(9.5, 10.7)	73	
GlcN-4	5.02, dd(9.4, 10.1)	70.4	
GlcN-5	3.94, m	69.5	
GlcN-6	4.25, dd(3.9, 12.5); 4.07, dd(1.9, 12.7)	63.6	
GlcN-NH	*6.03, d(9.4)	−	
GlcN-COCH_3_	−	174.1	
GlcN-COCH_3_	1.97, s	23.5	
Glyceroyl			
GroA-1	−	171.3	
GroA-2	4.69, m	76.7	
GroA-3	4.04, d(7.5); 3.80 d(10.0)	69.9	
Glycerol			
Gro-1	4.40, dd(3.1, 11.9); 4.15, dd(7.0, 11.9)	64.2	
Gro-2	5.22, m	72.2	
Gro-3	4.01, m	65.6	
Alkylamide			
RN-NH	*6.16, t(5.7)	−	
RN-1	3.2_3.3, m	41.2	
RN-2	1.59, m	31	
RN-3∼12	1.35-1.25, m	31.6-30.7	
RN-13		29	
RN-14	1.14, m	40.7	
RN-15	1.50, m	29.6	
RN-16,17	0.84, s	23.8	
Acyl			
RCO-1	−	175.4; 175.1	
RCO-2	2.30, t(7.4)	35.7; 35.6	
RCO-3	1.59, m	26.5; 26.6	
RCO-4	1.35-1.25, m	28.8	
RCO-5∼12(10)		31.6-30.7	
RCO-13(11)		28.9	
RCO-14(12)	1.14, m	40.7	
RCO-15(13)	1.50, m	29.6	
RCO-16, 17(14, 15)	0.84, d(6.7)	23.8	
Phosphate			−0.693

The spectra were measured in CD_3_OD/CDCl_3_ (1/1, v/v) at 303 K. The assignments were established by ^1^H, ^13^C and ^31^P one-dimensional spectrascopy, and the following two-dimensional methods: COSY, TOCSY, HMQC and HMBC, ^1^H-^31^P HMQC (*J*-constant: 8 Hz).

* Signals for the alkali-hydrolyzed and per-acetylated derivative in CDCl_3_.

By the modified TLC-blotting method, the yield of the glycolipids purified from the mixture was 40% on average. Compared to regular silica gel chromatography, the handling was much easier and the yield was comparable or higher especially for the acidic glycolipid.

### Structural Characterization of NGL-A

From the MALDI-TOF/MS spectrum of NGL-A, four remarkable peaks, m/z 1356.8, 1328.7, 1300.7, 1270.7, were detected in the positive mode measurement (Table 1). All four were mono-valent cation peaks. From the mass difference between the biggest peak at m/z 1328.7, it was suggested that they were congineers with micro-heterogeneity in the fatty acid part, which was also confirmed by the fatty acid analysis. The MALDI-TOF/MS spectrum of per-acetylated NGL-A showed four peaks at m/z 1734.7, 1706.6, 1678.6, 1648.7. The biggest peak was m/z 1706.6. By the per-acetylation, each peak was found to move to plus 378, suggesting that nine acetyl groups were incorporated to NGL-A by the per-acetylation reaction. This means that nine free hydroxyl groups exist in the NGL-A.

No phosphorus was detected in the composition analysis and no signal was found in ^31^P NMR, indicating that phosphate was not incorporated in the NGL-A. In the fatty acid analysis (Table 2), iso-heptadecanoic and iso-pentadecanoic acid were predominant. Galactose, glucose and glycerol were detected in the neutral sugar analysis. Glucosamine was only found in the amino acid analysis. No other amino acids were detected. Based on the glucosamine content, the molar ratio of each component was determined as shown in Table 2.

Since there were many overlapping peaks in the ^1^H-NMR of NGL-A in CDCl_3_/CD_3_OD (1/1, v/v), the assignment of peaks was done using per-acetylated NGL-A as summarized in Table 3. A trisaccharide was suggested from the three peaks around δ 100 in the ^13^C NMR. From the coupling constants (9.6 to 10 Hz) obtained for its *J*_2,3_, *J*_3,4_, *J*_4,5_, a glucose moiety was suggested. A galactose moiety was determined from the small coupling constant for its *J*_3,4_, *J*_4,5_. The multiplet signal at δ 2.96 of H-2 and doublet amide signal at δ 6.27 suggested a glucosamine moiety. All three moieties existed as pyranose forms. From the *J*_1,2_ values of each sugar moiety, α-glucoside (3.7 Hz), α-galactoside (3.6 Hz) and β-glucosamine (8.2 Hz) were characterized, respectively.

In the ^13^C NMR spectrum, 12 signals were detected around δ 170, indicating 3 carbonyl groups in the acyl moiety of the long fatty acids and 9 carbonyl groups in acetyl (Table 3). The ^1^H-^13^C HMBC spectrum showed the correlations between the 12 carbonyl carbons and appropriate protons, respectively (Fig. S1a). From these correlations, it was disclosed that the acetyl groups were attached to the 3, 4, and 6-OH groups of glucose, to the 3 and 4-OH groups of glucosamine, and to the 2, 3, 4, and 6-OH of galactose. Also, long fatty acids were confirmed to attach to the 1-OH of glycerol and the 2-NH of glucosamine. In addition, the ^1^H-^13^C HMBC spectrum (Fig. S1b) showed a correlation between the 1-C of glucose and the 3-H of glycerol, the 1-C of glucosamine and the 2-H of glucose, and the 1-C of galactose and the 6-H of glucosamine. Consequently, the rest of long fatty acid should be linked to the 2-OH of glycerol, although an appropriate cross peak between the carbonyl carbon of the long fatty acid and the 2-H of glycerol was not detected. From these results, the structure of NGL-A was determined as Gal(α1-6)GlcN(β1-2)Glc(α1-)acyl_2_Gro, as shown in Fig. 1a. The absolute configuration of the 2-position of glycerol has not been determined. Despite the micro-heterogeneity of the long fatty acid parts, a predominant example was shown in Fig. 2a, where the long fatty acids were two iso-pentadecanoic acids and one iso-heptadecanoic acid.

### Structural Characterization of NGL-C

Similar per-acetylation, MALDI-TOF/MS and NMR measurements were applied for NGL-C. In the positive mode MALDI-TOF/MS of NGL-C, molecular ion peaks were found at m/z 1519.0, 1491.1, 1463.0 and 1434.1. The peak at m/z 1491.1 was the biggest. Similar to NGL-A, these peaks were due to the micro-heterogeneity of the long fatty acid. By the per-acetylation of NGL-C, these 4 peaks were moved to m/z 2023.7, 1995.7, 1965.7 and 1937.6 (Table 1). The biggest was m/z 1995.7. The increment of 504 for each peak suggested that 12 units of acetyl moieties were incorporated to NGL-C by the per-acetylation. Compared to NGL-A, the difference in molecular peaks between the biggest ones was 162, suggesting an additional hexose in NGL-C. From the hexose content by GC analysis, the ratio of galactose to glucose was 2∶ 1, suggesting that the additional hexose was galactose. No signal was detected in the ^31^P NMR spectrum. Due to many overlapping proton peaks in the ^1^H NMR spectra of NGL-C itself, all protons of NGL-C were assigned by ^1^H and ^13^C NMR using per-acetylated NGL-C (Table 3).

From ^13^C NMR, the 4 peaks around δ 100 suggested a tetra-saccharide. One of them appeared at a lower field (δ 107), suggesting a furanose structure. From the COSY and TOCSY spectra, protons belonging to saccharide moieties were assigned. Chemical shift values of 4-H and 5-H suggested that the glucose and the glucosamine were in pyranose form, and the two galactoses were in furanose and pyranose form, respectively. Based on coupling constants between 1-H and 2-H, α-glucopyranose (*J*_1,2_ = 3.6 Hz) and α-galactopyranose (*J*_1,2_ = 3.4 Hz) were elucidated. The glucosamine was β form (*J*_1,2_ = 8.1 Hz). The galactofuranose was also β-form because of a singlet signal at 1-H.

In the ^13^C NMR of per-acetylated NGL-C, 15 carbonyl peaks around δ 170 were found; 3 were related to long fatty acids and the rest were acetyl groups. From the correlation between these carbonyl peaks and protons in the saccharide moieties in the ^1^H-^13^C HMBC spectrum (Fig. S2a), the positions of the acetyl groups were determined. Basically, the NMR spectra of per-acetylated NGL-C were similar to those of NGL-A, except for the additional galactofuranose. In this galactose moiety, the hydroxy group at the 5 position was acetylated, confirming the furanose form. This ^1^H-^13^C HMBC spectrum also showed correlations between the 1-C of glucose and the 3-H of glycerol, the 1-C of glucosamine and the 2-H of glucose, the 1-C of galactopyranose and the 6-H of glucosamine, and the 1-C of galactofuranose and the 2-H of galactopyranose. Reverse correlations, such as the 1-H of glucose and the 3-C of glycerol, were also found (Fig. S2b). From these facts, the structure of NGL-C was determined as Gal*f*(β1-2)Gal*p*(α1-6)Glc*pN*-acyl (β1-2)Glc*p*(α1-3)acyl_2_Gro (Fig. 1b). Although the absolute configuration of the 2-position of glycerol has not been determined, this structure was coincident to that reported previously by Oshima *et al.* [19]. They reported that the main long fatty acid linked to an amino group in glucosamine was iso-heptadecanoic acid. In our MALDI-TOF/MS analysis, the biggest peak (m/z = 1491.1) indicates the molecular ion peak [M+Na]^+^, where M involves two iso-heptadecanoic acids and one iso-pentadecanoic acid.

### Structural Characterization of Acidic Glycolipid AGL-B

In the negative mode EST-TOF/MS spectrum of AGL-B, molecular ion peaks, at m/z 1176.0 and 1204.0, were detected (Table 1). From the mass difference between these peaks, it was suggested that they are congeners having micro-heterogeneity in the fatty acid part as shown in NGL-A and NGL-C. By the per-acetylation, remarkable peaks were shifted to m/z 1302.0 and 1329.9. This suggests that three acetyl groups were incorporated into AGL-B by the per-acetylation reaction, resulting in three free hydroxyl groups in AGL-B.

From the composition analyses (Table 1), phosphorus was found, suggesting the presence of phosphate. The same types of long fatty acids were detected, again, the predominant ones being iso-heptadecanoic and iso-pentadecanoic acid. Glycerol and glucosamine moieties were confirmed, but no glucose or galactose was found. The relative molar ratio of glucosamine/glycerol/phosphorus/fatty acid was 1/1/1/2. However, the combination of these components had a lower molecular weight than that found in MS, suggesting other unknown component(s) involved in AGL-B.

In the NMR spectra of AGL-B itself and per-acetylated AGL-B in CDCl_3_/CD_3_OD, the many overlapping proton peaks made full assignment impossible, therefore, a chemical decomposition was applied. In the ESI-TOF/MS spectrum of the alkali-hydrolyzed product of AGL-B, several peaks were detected at m/z 547.3 ([M+H]^+^), 569.2 ([M+Na]^+^) and 1115.5 ([2M+Na]^+^). No peak was found in the ^31^P NMR of the hydrolyzed product, suggesting cleavage of the phosphodiester group in the AGL-B. The COSY spectra are shown in Fig. S3. Protons in the glucosamine moiety and in the structure of -C(O)H-C(O)H_2_- were confirmed. The glucosamine was α-pyranose (*J*_1,2_ = 3.4 Hz). Acetyl protons were found at δ 2.0, suggesting *N*-acetyl glucosamine. No protons related to the glycerol were found. Although this product was obtained by alkali-hydrolysis, an iso-type long alkyl group was detected, suggesting that this long alkyl group was not due to an acyl group. From the lower chemical shift value (δ 3.2 ∼ 3.3) of the peripheral methyl group, it was disclosed that this alkyl group formed alkylamine and attached to the above -C(O)H-C(O)H_2_- group *via* an amide bond.

Further per-acetylation was applied to the alkali-hydrolyzed product. In the positive mode ESI-TOF/MS measurement, a molecular ion peak was found at m/z 737 as [M+Na]^+^ , suggesting the introduction of four acetyl groups. Compared to the per-acetylation of the original AGL-B, the additional acetyl group was suggested to have been introduced at the OH group which was originally linked to the phosphate group. Based on COSY spectra (Fig. S3), the full assignment of this per-acetylated compound was done and is summarized in Table 4. Amide protons were detected at δ 6.16 (triplet) and 6.02 (doublet), respectively. The triplet peak at δ 6.16 was due to the long alkyl group, and the doublet peak (δ 6.02) was related to the *N*-acetyl group in the glucosamine moiety. These assignments were further confirmed by TOCSY measurement (Fig. S4). From the ^1^H-^13^C HMBC spectrum (Fig. S5a), the carbonyl carbon at δ 166.7 was correlated to the proton in -C(O)H-C(O)H_2_-, suggesting the presence of a glyceric acid. In addition, this carbonyl carbon was correlated to protons of the peripheral methylene and amino group in the long alkyl amino group. From the integral of 1D ^1^H-NMR spectrum and MS data, the long alkyl amino group was found to be an iso-heptadecanolyamino group. The positions of the incorporated acetyl groups were all determined. Glucosamine was attached to the 3-position of glyceric acid (Fig. S5b). From these data including MS, the structure of the per-acetylated compound of alkali-hydrolyzed product was determined as shown in Fig. S6.

According to the partial structure of AGL-B, the full assignment of per-acetylated AGL-B was done as shown in Table 5. In the ^13^C-NMR spectrum, only one anomeric carbon (δ 98) was found, suggesting a monosaccharide (glucosamine) derivative. From the COSY spectra (Fig. S7), all protons in glucosamine, glycerol, glyceric acid, alkylamine and long fatty acids were determined. From the correlations around the region of the carbonyl carbon in the ^1^H-^13^C HMBC spectrum (Fig. S8), acetyl groups were confirmed to attach to the 3-, 4- and 6-OH of glucosamine. Also, correlations between the carbonyl carbon (δ 171.3) of glyceric acid and the 3-H (δ 3.80) of the glyceric acid and between the carbonyl carbon and the peripheral methylene protons (δ 3.2∼3.3) of alkyl amino group were found. Two carbonyl carbons of long fatty acids were detected, one of which was correlated to the 1-H of glycerol. Although correlation of the rest of the 2-H in the glycerol was not detected because of overlapping with HDO, it was assumed to form a diacyl glycerol structure. The ^1^H-^31^P HMBC spectrum shows the correlation between the phosphorus and the 3-H of glycerol, and the phophorous and the 3-H of glyceric acid. Correlation with the 2-H of the glyceric acid was not detected because of overlapping with HDO. However, since the 3-O of the glyceric acid was linked to glucosamine, it was concluded that the rest of the 2-OH of glyceric acid was consequently linked to phosphate. From these, the strucure of AGL-B was determined as shown in Fig. 2. The absolute configuration of the 2-position of glycerol was determined enzymatically. Since an alkali phosphatase treatment gave the molecular ion (m/z 569.41, [M+Na]^+^) corresponding to the deacylated AGL-B, the *sn*-configuration was determined. The 2-position of the glyceric acid was determined as R by the modified Mocher method [19] because of the positive value of Δδ (δ_S_ – δ_R_). From the composition analysis, micro-heterogeneity of the fatty acid was indicated. If the predominant iso-pentadecanoic and iso-heptadecanoic acids were the two fatty acids, the resulting molecular mass (1176.9) was the same as that observed (m/z 1176.0, [M–H]^–^).

## Discussion

The present study elucidated the structure of the acidic glycolipid (AGL-B), which may be the first report regarding the structure of the acidic glycolipid from *T. thermophilus* HB8. A similar glycophosholipid was reported from radiation resistant Gram-positive *Deinococcus radiodurans* (7–9). Its scaffold was *N*-(((Glc*p*NAc(α1-)acyl_2_Gro)P-2)GroA)alkylamine, where the predominant long alkyl groups in the glycophospholipid were non-branch type. According to the reports, the glucosamine was sometimes replaced by glucose. They also reported that the absolute configuration of phosphatidic acid was 1,2-diacyl-*sn*-glycerol 3-phosphate, and D-glyceric acid. These are also the same as those of AGL-B.

The biological significance of the glycophospholipid of *T. thermophirus* is not clear at the moment. It would be an interesting subject to study the reason why thermal stable and radiation-resistant bacteria share the same scaffold for their glycophospholipids on the surface and why the iso-type alkyl groups exist in only thermal stable bacteria. In *Thermus* species, common properties are presented regarding neutral glycolipids [20]–[24]. Despite the diversity of sugar moieties included in the neutral glycolipids, the scaffold of diacyl-diglycosyl-(*N*-acyl)-glucosaminyl-glycosyl-glycerol or diacyl-glycosyl-(*N*-acyl)-glucosaminyl-glycosyl-glycerol is common. The structures of NGL-A and NGL-C in the present study belong to this family. It was emphasized that the predominant fatty acid in the family of neutral glycolipids was iso-pentadecanoyl or iso-heptadecanoyl acid, and the relative contents of these iso-type fatty acid increased with increasing the temperature during cultivation [24]. In the structure of the novel acidic glycolipid (AGL-B), of *T. thermophilus* HB8, the fatty acids and the long alkyl group were predominantly iso-type. We also confirmed that no lipopolysaccharide (LPS) exists in *T. thermophilus* HB8 (data not shown). From these facts, it is speculated that the iso-type long alkyl group plays a crucial role in the stability of the membrane of this bacterium. The study to elucidate the biosynthetic pathway of AGL-B is underway in combination with its total chemical synthesis.

## Supporting Information

Figure S1 **^1^H-^13^C HMBC spectra of per-acetylated NGL-A.** (a) Region around the carbonyl groups; (b) around the glycoside linking region.
(PDF)

Figure S2 **^1^H-^13^C HMBC spectra of per-acetylated NGL-C.** (a) Region around the carbonyl groups; (b) around the glycoside linking region.
(PDF)

Figure S3 **COSY spectra of the alkali-hydrolyzed and per-acetylated product from the acidic glycolipid (AGL-B) from** ***T. thermophilus*** **HB8.** (a) Region around the sugar moieties; (b) region around the alkyl moieties.
(PDF)

Figure S4 **TOCSY spectrum (amide region) of the alkali-hydrolyzed and per-acetylated product from the acidic glycolipid (AGL-B) from** ***T. thermophilus*** **HB8.**
(PDF)

Figure S5 **^1^H-^13^C HMBC spectra of the alkali-hydrolyzed and per-acetylated product from the acidic glycolipid (AGL-B) from** ***T. thermophilus*** **HB8.** (a) Region around the carbonyl groups; (b) around the glycoside linkage region.
(PDF)

Figure S6 **Proposed structure of the alkali-hydrolyzed and per-acetylated product from the acidic glycolipid (AGL-B) from** ***T. thermophilus*** **HB8.**
(PDF)

Figure S7 **COSY spectra of the per-acetylated acidic glycolipid (AGL-B) from** ***T. thermophilus*** **HB8.** (a) Region around the sugar moieties; (b) region around the alkyl moieties.
(PDF)

Figure S8 **^1^H-^13^C HMBC spectra of the per-acetylated acidic glycolipid (AGL-B) from** ***T. thermophilus*** **HB8.** (a) Region around the carbonyl groups; (b) full region.
(PDF)

## References

[1] Oshima T, Imahahori K (1974). Description of *Thermus thermophilus* (Yoshida and Oshima) comb. nov., a Nonsporulating Thermophilic Bacterium from a Japanese Thermal Spa.. Int J Syst Bacteriol.

[2] Stallings WC, Pattridge KA, Strong RK, Ludwig ML (1985). The structure of manganese superoxide dismutase from *Thermus thermophilus* HB8 at 2.4- Å resolution.. J Biol Chem.

[3] Imada K, Sato M, Tanaka N, Katsube Y, Matsuura Y (1991). Three-dimensional structure of a highly thermostable enzyme, 3-isopropylmalate dehydrogenase of *Thermus thermophilus* at 2.2Å resolution.. J Mol Biol.

[4] Fujinaga M, Berthet-Colominas C, Yaremchuk AD, Tukalo MA, Cusack S (1993). Refined Crystal Structure of the Seryl-tRNA Synthetase from *Thermus thermophilus* at 2–5 Å Resolustion.. J Mol Biol.

[5] Kuramitsu S, Kawaguchi S, Hiramatsu Y (1995). Database of heat-stable proteins from *Thermus thermophilus* HB8.. Protein Eng.

[6] Yokoyama S, Matsuo Y, Hirota H, Kigawa T, Shirouzu M (2000). Structural genomics projects in Japan.. Prog Biophys Mol Biol.

[7] Anderson R, Hansen K (1985). Structure of a Novel Phosphoglycolipid from *Deinococcus radiodurans*.. J Biol Chem.

[8] Huang Y, Anderson R (1989). Structure of a Novel Glucosamine-containing Phosphoglycolipid from *Deinococcus radiodurans*.. J Biol Chem.

[9] Huang Y, Anderson R (1991). Phosphatidylglyceroylalkylamine, a novel phosphoglycolipid precursor in *Deinococcus radiodurans*.. J Bacteriol.

[10] Boone DR, Castenholz RW, Garrity GM, editors (2001). Bergey’s Manual of Systematic Bacteriology 2nd.. ed., vol.1, Springer, 396.

[11] Bartlett GR (1959). Phosphorus Assay in Column Chromatography.. J Biol Chem.

[12] Ashwell G (1957). Colorimetric analysis of sugar. Methods in Enzymology..

[13] Torello LA, Yates AJ, Thompson DK (1980). Critical study of the alditol acetate method for quantitating small quantities of hexoses and hexosamines in gangliosides.. J Chromotgr.

[14] Suda Y, Tochio H, Kawano K, Takada H, Yoshida T (1995). Cytokine-inducing glycolipids in the lipoteichoic acid fraction from *Enterococcus hirae* ATCC 9790.. FEMS Immun Med Microbiol.

[15] Ikemoto S, Katoh K, Komagata K (1978). Cellular fatty acid composition in methanol-utilizing bacteria.. J Gen Appl Microbiol.

[16] Hashimoto M, Kirikae F, Dohi T, Kusumoto S, Suda Y (2001). Structural elucidation of a capsular polysaccharide from a clinical isolate of *Bacteroides vulgatus* from a patient with Crohn’s disease.. Eur J Biochem.

[17] Taki T, Kasama T, Handa S, Ishikawa D (1994). A Simple and Quantitative Purification of Glycosphingolipids and Phospholipids by Thin-layer Chromatography Blotting.. Anal Biochem.

[18] Ohtani I, Kusumi T, Kashman Y, Kakisawa H (1991). High-field FT NMR application of Mosher’s method. The absolute configurations of marine terpenoids.. J Am Chem Soc.

[19] Oshima M, Ariga T (1976). Analysis of the anomeric configuration of a galactofuranose containing glycolipid from an extreme thermophile.. FEBS Lett.

[20] De Haas GH, Postema NM, Nieuwenhuizen W, Van Deenen LLM (1968). Purification and properties of phospholipase A from porcine pancreas.. Biochim Biophys Acta.

[21] Pask-Hughes AR, Shaw N (1982). Glycolipids from Some Extreme Thermophilic Bacteria Belonging to the Genus *Thermus*.. J Bacteriol.

[22] Wait R, Caretto L, Nobre MF, Ferreira AM, Da Costa MS (1997). Characterization of novel long-chain 1,2-diols in *Thermus* species and demonstration that *Thermus* strains contain both glycerol-linked and diol-linked glycolipids.. J Bacteriol.

[23] Prado A, Da Costa MS, Laynez J, Medeira VM (1988). Physical properties of membrane lipids isolated from a thermophilic eubacterium (*Thermus* sp.).. Adv Exp Med Biol.

[24] Ray PH, White DC, Brock TD (1971). Effect of Growth Temperature on the Lipid Composition of *Thermus aquaticus*.. J Bacteriol.

