# Supplementary material for: Structural Characterization of Neutral and Acidic Glycolipids from Thermus thermophilus HB8
Source: PLoS One. 2012 Jul 16;7(7):e35067. doi: 10.1371/journal.pone.0035067 (PMC3398001; doi:10.1371/journal.pone.0035067)
Supplement: Figure S1 — 1H-13C HMBC spectra of per-acetylated NGL-A. (a) Region around the carbonyl groups; (b) around the glycoside linking region. (PDF) [file pone.0035067.s001.pdf]

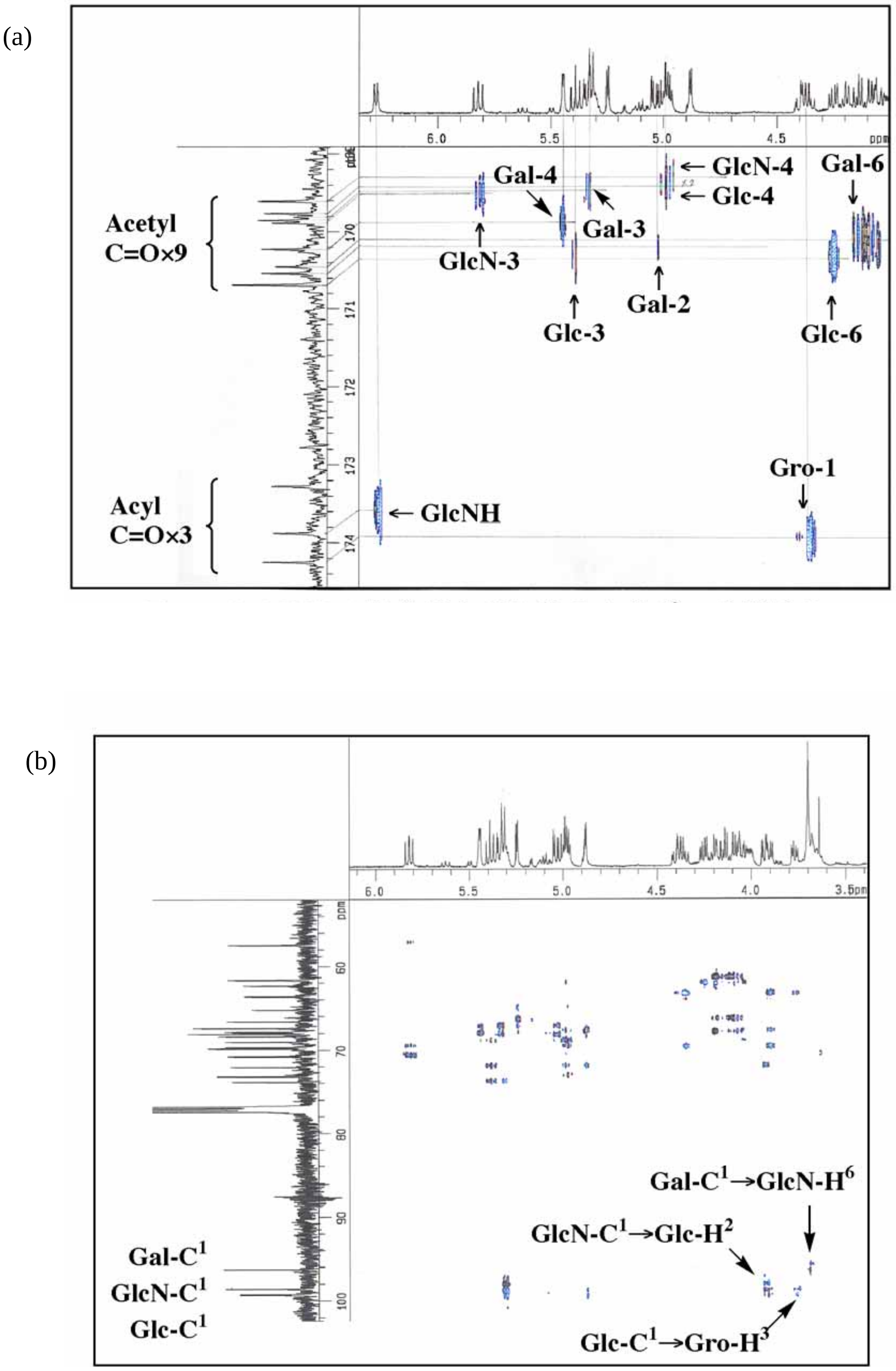

Figure S1 ^{1}H-^{13}C HMBC spectra of per-acetylated NGL-A. (a) Region around the carbonyl groups; (b) around the glycoside linking region.
