# Supplementary material for: Structural Characterization of Neutral and Acidic Glycolipids from Thermus thermophilus HB8
Source: PLoS One. 2012 Jul 16;7(7):e35067. doi: 10.1371/journal.pone.0035067 (PMC3398001; doi:10.1371/journal.pone.0035067)
Supplement: Figure S6 — Proposed structure of the alkali-hydrolyzed and per-acetylated product from the acidic glycolipid (AGL-B) from T. thermophilus HB8. (PDF) [file pone.0035067.s006.pdf]

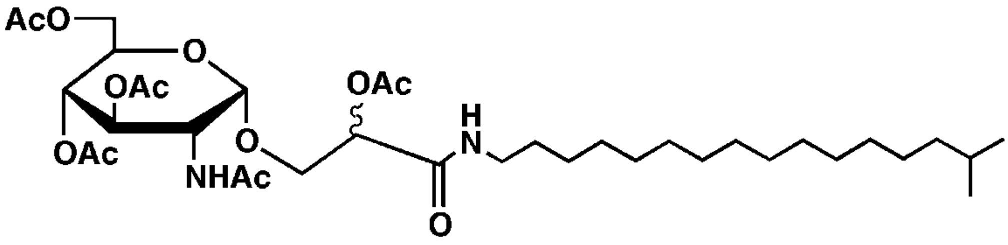

Figure S6 Proposed structure of the alkali-hydrolyzed and per-acetylated product from the acidic glycolipid (AGL-B) from *T. thermophilus* HB8.
