# Supplementary material for: Structural Characterization of Neutral and Acidic Glycolipids from Thermus thermophilus HB8
Source: PLoS One. 2012 Jul 16;7(7):e35067. doi: 10.1371/journal.pone.0035067 (PMC3398001; doi:10.1371/journal.pone.0035067)
Supplement: Figure S7 — COSY spectra of the per-acetylated acidic glycolipid (AGL-B) from T. thermophilus HB8. (a) Region around the sugar moieties; (b) region around the alkyl moieties. (PDF) [file pone.0035067.s007.pdf]

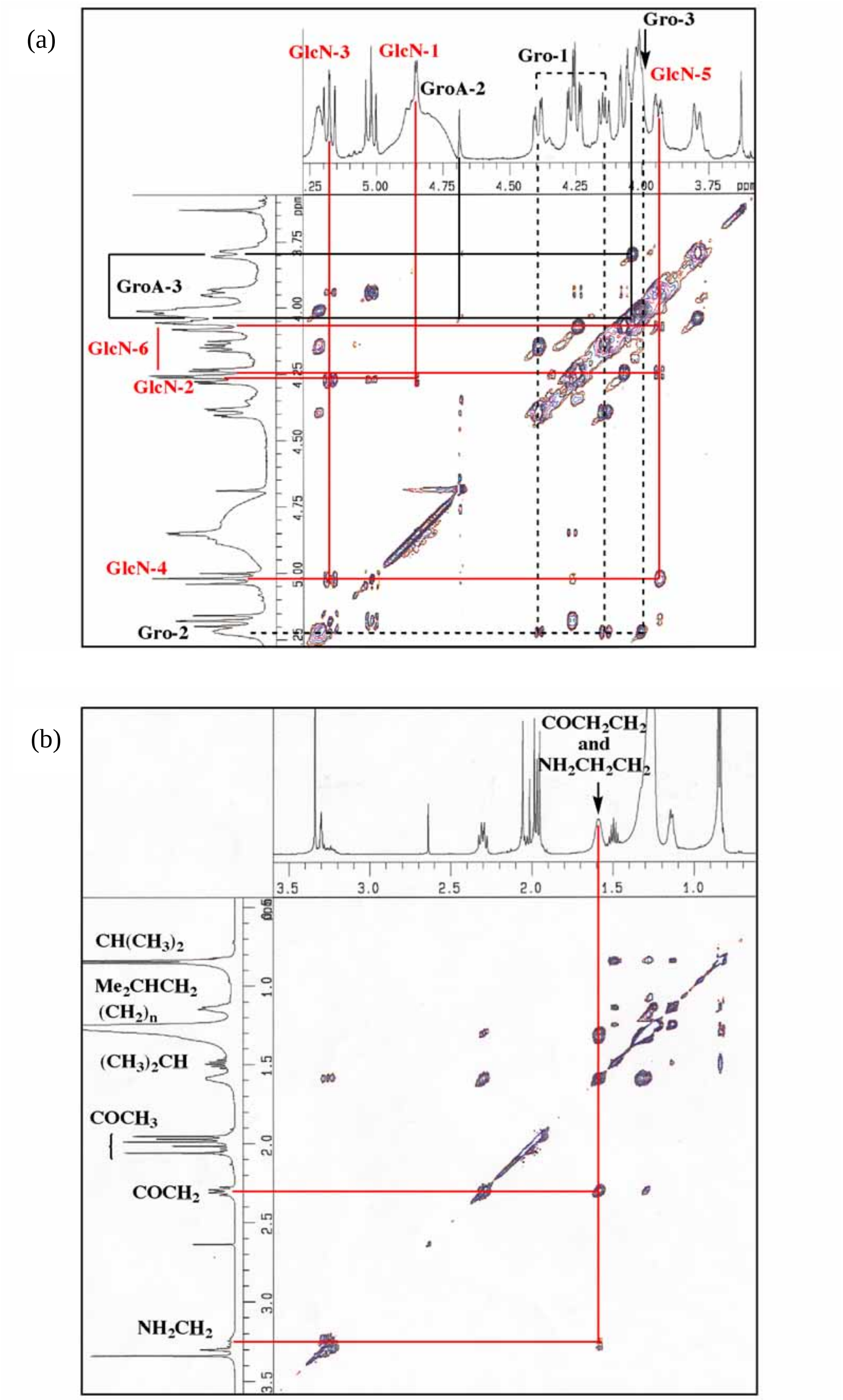

Figure S7 COSY spectra of the per-acetylated acidic glycolipid (AGL-B) from *T. thermophilus* HB8. (a) Region around the sugar moieties; (b) region around the alkyl moieties.
