# Supplementary material for: Structural Characterization of Neutral and Acidic Glycolipids from Thermus thermophilus HB8
Source: PLoS One. 2012 Jul 16;7(7):e35067. doi: 10.1371/journal.pone.0035067 (PMC3398001; doi:10.1371/journal.pone.0035067)
Supplement: Figure S8 — 1H-13C HMBC spectra of the per-acetylated acidic glycolipid (AGL-B) from T. thermophilus HB8. (a) Region around the carbonyl groups; (b) full region. (PDF) [file pone.0035067.s008.pdf]

(a)

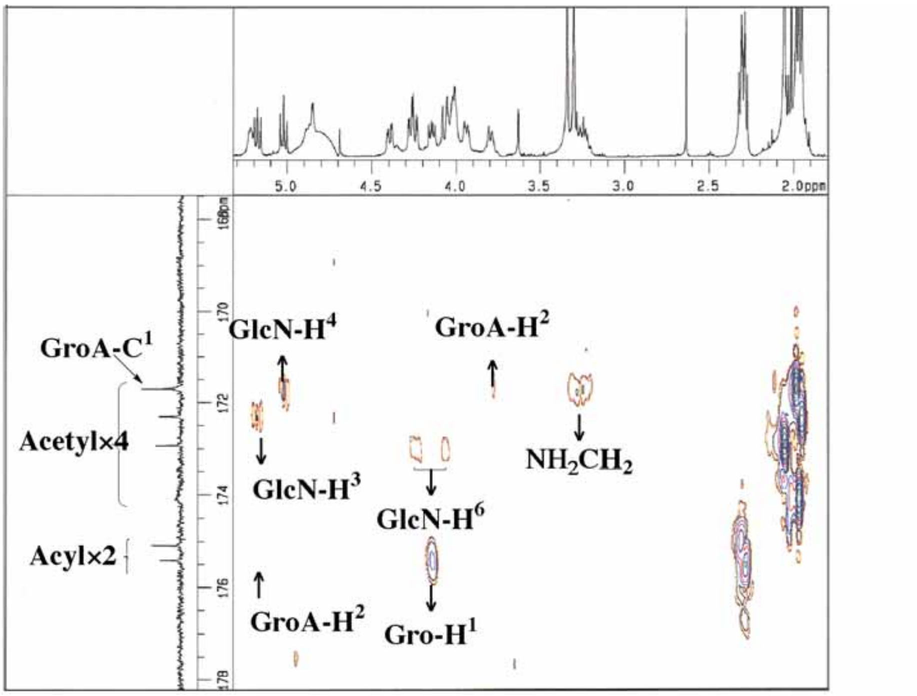

(b)

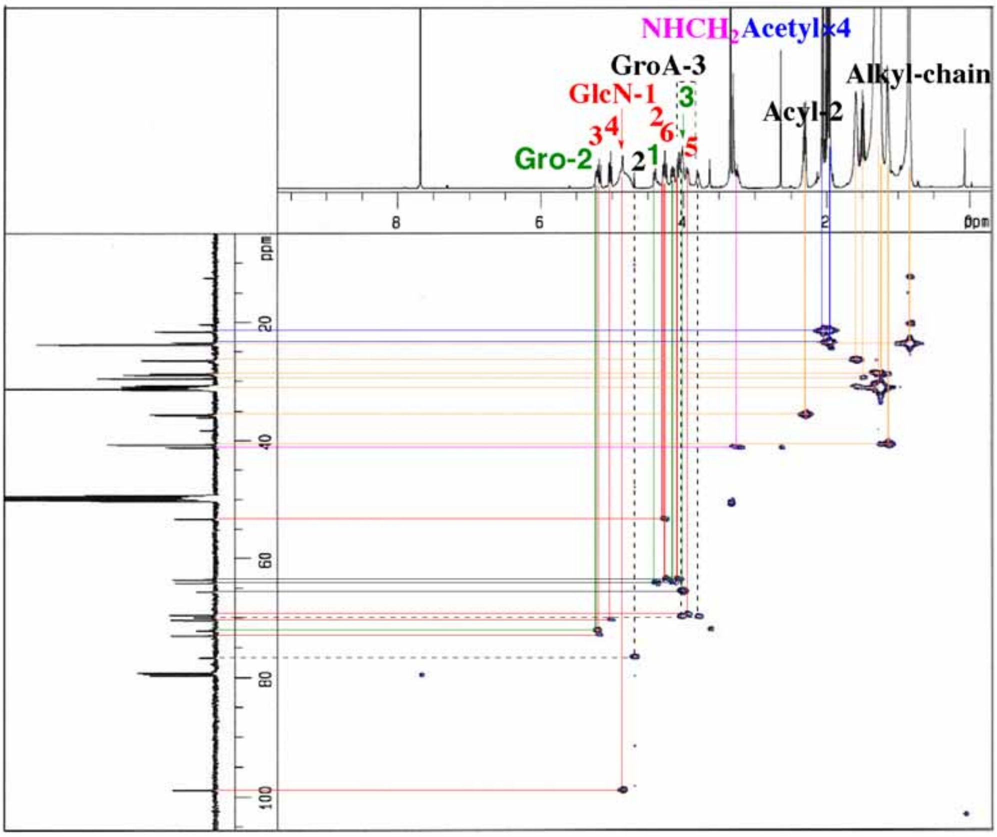

Figure S8 ^{1}H-^{13}C HMBC spectra of the per-acetylated acidic glycolipid (AGL-B) from *T. thermophilus* HB8. (a) Region around the carbonyl groups; (b) full region.
